# Characterization of the gut microbiota in the golden takin (*Budorcas taxicolor bedfordi*)

**DOI:** 10.1186/s13568-017-0374-5

**Published:** 2017-04-17

**Authors:** Jun Chen, Huanxin Zhang, Xiaoyang Wu, Shuai Shang, Jiakuo Yan, Yao Chen, Honghai Zhang, Xuexi Tang

**Affiliations:** 1 College of Life Science, Qufu Normal University, Jing xuan West Street No. 57, Qufu, 273165 Shandong People’s Republic of China; 2 College of Marine Life Sciences, Ocean University of China, Qingdao, China

**Keywords:** Gut microbiota, 16SrRNA, Illumina sequencing, Seasonal changes, Diet quality, Golden takin (*B. t. bedfordi*)

## Abstract

The gut microbiota of mammals is a complex ecosystem, which is essential for maintaining gut homeostasis and the host’s health. The high throughput sequencing allowed us to gain a deeper insight into the bacterial structure and diversity. In order to improve the health status of the endangered golden takins, we first characterized the fecal microbiota of healthy golden takins using high throughput sequencing of the 16S rRNA genes V3–V4 hypervariable regions. Our results showed that, Firstly, the gut microbiota community comprised 21 phyla, 40 classes, 62 orders, 96 families, and 216 genera. *Firmicutes* (67.59%) was the most abundant phylum, followed by *Bacteroidetes* (23.57%) and *Proteobacteria* (2.37%). Secondly, the golden takin maintained higher richness in spring than in the winter while community diversity and evenness was not significantly different. Thirdly, four female golden takins demonstrated highly similar microbiota and the five golden takin males had relatively highly similar microbiota. All of our results might indicate that the fecal microbiota of golden takins were influenced by the season and the animal’s sex. The findings provided theoretical basis regarding the gut microbiota of golden takins and may offer new insights to protect this endangered species.

## Introduction

The takin (*Budorcas taxicolor*) is a large Himalayan ungulate in the family Bovidae, which is classified into four subspecies based on distinct differences in physical characteristics and geographic location, including the golden takin (*B. t.bedfordi*), the Mishmi takin (*B. t.taxicolor*), the Sichuan takin (*B. t. Tibetana*), and the Bhutan takin (*B. t.whitei*). The golden takin and the Sichuan takin are endemic subspecies of China. Furthermore, relative to the other subspecies, the golden takin is listed as vulnerable by the International Union for Conservation of Nature (IUCN 2008) and is under state protection (category I) in China. The golden takin is a type of social bovid herbivore. As a ruminant, this animal primarily forages on the browse of trees and shrubs (Schaller et al. 1986). In the spring and summer, golden takins undertake seasonal migration to the higher elevations of the uppermost tree line. During the autumn and winter, they move to lower elevations with smaller groups (Ming et al. 2003).

The gut microbiota offers substantial benefits to the host, such as aiding in digestion, promoting immune system development and competing for niches with pathogens (Cadwell 2014). To date, there have been many studies investigating the gut microbiota in herbivores (Bian et al. 2013; Costa et al. 2012; Liu et al. 2014). For instance, *Firmicutes* and *Bacteroidetes*, represented by the *Ruminococcaceae*, *Lachnospiraceae*, *Rikenellaceae* and *Prevotellaceae* families (Bian et al. 2013), dominate the microbiota in the healthy rhinoceros. Costa et al. (2012) described the predominance of *Firmicutes* (68%) in the feces of healthy horses, followed by *Bacteroidetes* (14%) and *Proteobacteria* (10%). The core microbiota of donkeys is dominated by *Firmicutes* (64% males and 64% females) and *Bacteroidetes* (23% males and 21% females), followed by *Verrucomicrobia*, *Euryarchaeota*, *Spirochaetes* and *Proteobacteria* (Liu et al. 2014). Considerable evidence supports the complexity of the gut microbiota in many mammals and the necessity of gut bacterial communities to maintain gut homeostasis. However, surprisingly, little research was on the gut microbiota of the golden takin. Therefore, in present research, we characterized the fecal microbiota of nine golden takins by performing high-throughput sequencing. On this basis, we also compared gut microbiota variation from winter to spring, which is the season of animals likely to get sick. Our result indicated that season was a factor that might impact the composition of the gut microbiota.

## Materials and methods

### Animal selection

Nine healthy golden takins were enrolled in this study (Table 1). Four fresh fecal samples (Group A) were collected at Jinan wild animal park during the late morning in December 2015. Five fresh fecal samples (Group B) were collected at Jinan Animal Park during the late morning in April 2016. Group A and group B respectively represented the winter and spring feces samples. All of the animals were about 8 years old and the two environments were roughly same. The experiment was approved by the Qufu Normal University Animal Care and Use Committee. None of the animals had received anti-inflammatory drugs or antimicrobials within the prior 4 months, and none had a gastrointestinal-related disease. Samples were collected off the ground immediately after defecation, and each sample was instantly transferred into plastic tubes. The samples were stored at 4 °C with long-term storage at −80 °C for further analysis.

**Table 1:** Information on the characteristics of the golden takins used in this study

Group	Golden takin	Sex	Place
A	J1	Female	Jinan wild animal park
	J2	Male	Jinan wild animal park
	J3	Male	Jinan wild animal park
	J4	Male	Jinan wild animal park
B	J5	Female	Jinan animal park
	J6	Male	Jinan animal park
	J7	Male	Jinan animal park
	J8	Female	Jinan animal park
	J9	Female	Jinan animal park

### DNA extraction, 16S rRNA gene PCR and sequencing

Total genomic DNA was isolated from fecal samples using a QIAamp DNA Stool Mini Kit (Qiagen, Germany) according to the manufacturer’s recommended protocol. We used a UV–Vis spectrophotometer (NanoDrop 2000c, USA) to determine DNA quantification and quality. DNA concentrations were measured to ensure final concentrations greater than 20 ng/μl for PCR amplification of the V3–V4 hypervariable regions of the 16SrRNA gene using the primers 341F (CCTAYGGGRBGCASCAG) and 806R (GGACTACNNGGGTATCTAAT), as previously described (Hugerth et al. 2014). Amplification was performed in a 30-μl volume, and the amplified fragment size was approximately 410 bp. Specifically, PCR reactions contained 15 μl of Phusion Master Mix (2er), 1.5 µl of each primer, 10 µl of microbial genomic DNA, and 2 µl ddH_2_O to constitute a final volume of 30 µl. The following PCR protocol was used: 1 min at 98 °C for initial denaturation, 35 cycles of 10 s at 98 °C for denaturation, 30 s at 55 °C for annealing, and 30 s at 72 °C for elongation, and a final extension at 72 °C for 5 min. PCR products were mixed with same volume of 1× loading buffer (containing SYBR green) and separated by electrophoresis on a 2% agarose gel. Samples containing a bright band between 400 and 450 bp were chosen and mixed in equidensity ratios. Then, this mixture of PCR products was purified with a Qiagen Gel Extraction Kit (Qiagen, Germany). Sequencing libraries were generated using a TruSeq^®^ DNA PCR-Free Sample Preparation Kit (Illumina, USA) following the manufacturer’s recommendations, and index codes were added. Library quality was assessed with a Qubit@ 2.0 Fluorometer (Thermo Scientific) and an Agilent Bioanalyzer 2100 system. Finally, the DNA library was sequenced on an IlluminaHiSeq 2500 platform, and 250-bp paired-end reads were generated.

### Bioinformatics analysis

Paired-end reads were assigned to samples based on their unique barcodes and truncated by cutting off the barcodes and primer sequences. Paired-end reads were then merged using FLASH (V1.2.7) (Magoč and Salzberg 2011). The raw tags were subjected to specific filtering conditions to obtain high-quality clean tags using the QIIME (V1.7.0) (Caporaso et al. 2010) quality control process. The tags were compared with the reference database (Gold database) using the UCHIME algorithm (UCHIME Algorithm) (Edgar et al. 2011) to detect chimeric sequences, which were then removed. Finally, effective tags were obtained. Sequence analysis was performed using Uparse software (Uparse v7.0.1001) (Edgar 2013). Sequences demonstrating greater than or equal to 97% similarity were assigned to the same operational taxonomic unit (OTU). For each representative sequence, the GreenGene Database (Desantis et al. 2006) was used and the RDP classifier algorithm (Version 2.2) (Wang et al. 2007) was employed to annotate taxonomic information. To study the phylogenetic relationships between different OTUs and identify dominant species in different samples (groups), multiple sequence alignment was carried out using MUSCLE software (Version 3.8.31) (Edgar 2004). Ultimately, we obtained 54,748 sequences for diversity analysis. Alpha diversity was evaluated by calculating six indices, specifically the Observed-species, Chao1, Shannon, Simpson, ACE and Good-coverage indices. All indices were calculated with QIIME (V1.7.0) and displayed with R software (Version 2.15.3). Beta diversity based on both Weighted and Unweighted Unifrac distances was calculated using QIIME software (Version 1.7.0). Cluster analysis was preceded by principal component analysis (PCA) using the FactoMineR package and ggplot2 package in R software (Version 2.15.3). The data set supporting the results of this article is available in the Sequencing Read Archive (SRA) database, accession numbers SRP090712.

## Results

**Fig. 1:**
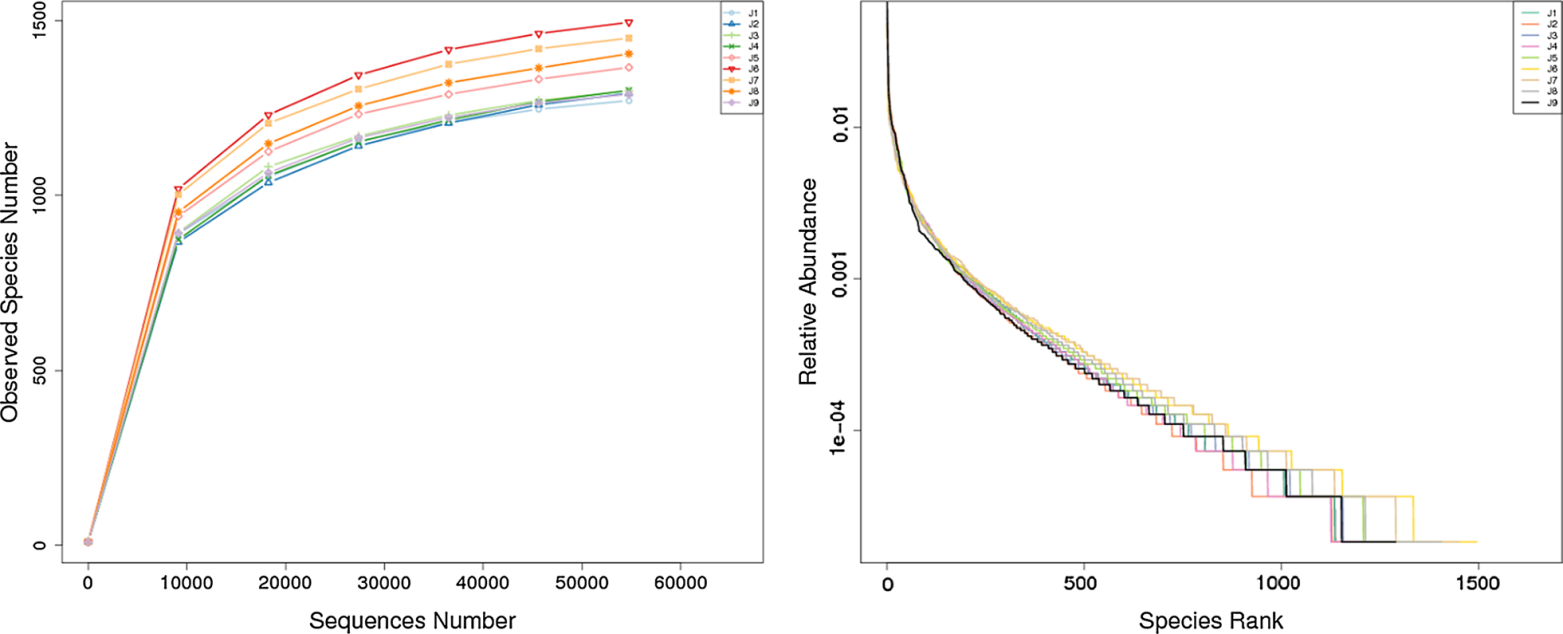
Rarefaction curve analysis of nine golden takins. Repeated samples of OTU subsets were performed to evaluate whether further sampling would likely yield additional taxa, as indicated by whether the curve reached a plateau value. The* y-axis* indicates the number of OTUs detected, and the *x-axis* indicates the number of taxa in the sequence subset analyzed. Rank abundance curves were used to estimate the richness and evenness of taxa present in the samples. The* y-axis* indicates the relative abundance of OTUs, and the *x-axis* indicates the number of OTUs according to the relative abundance from large to small. The larger the span curve on the* x-axis*, the higher the species richness. The smoother the curve on the* y-axis*, the more even the species distribution

**Fig. 2:**
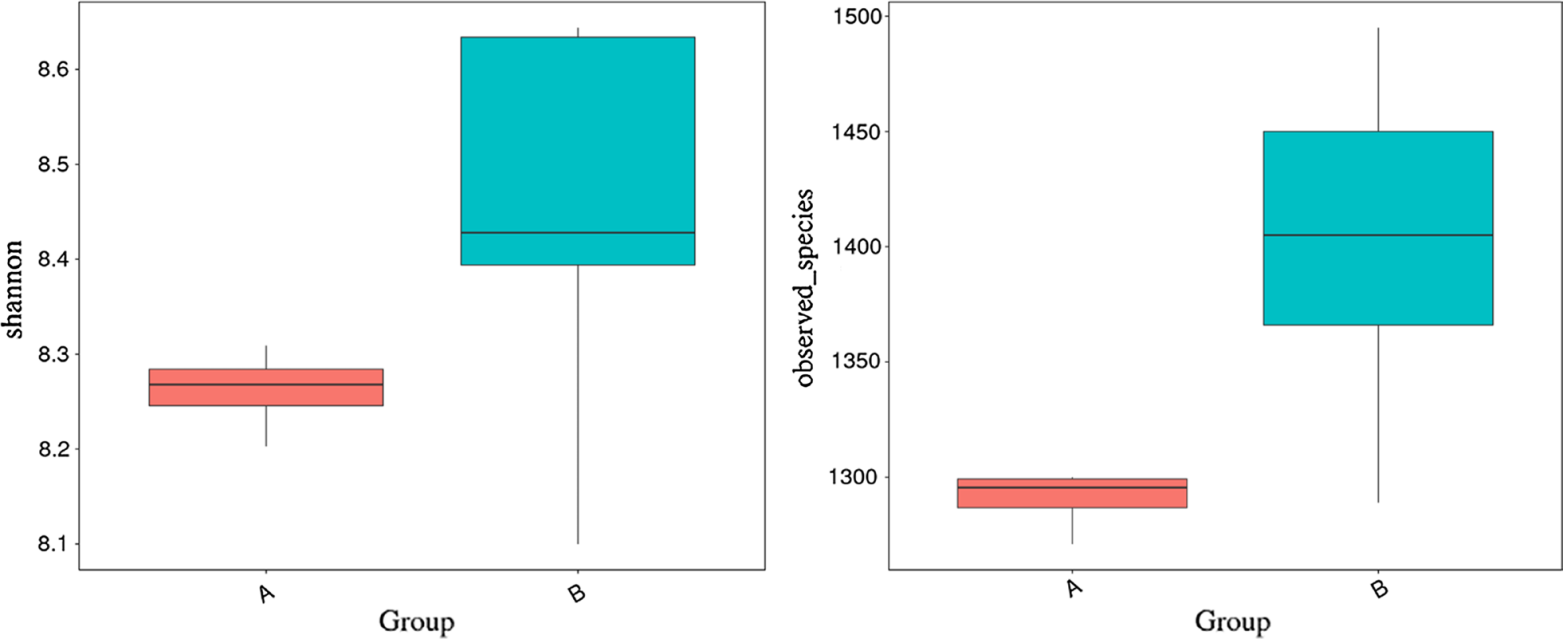
A comparison of community alpha diversities between winter and spring samples in golden takins. Diversity was measured by calculating the Shannon index, and richness was determined by calculating the Observed-species index

During quality control, a total of 670,356 high-quality sequences with an average length of 410 bp per sample were obtained from the nine fecal samples. The statistical estimates of species richness for the sequences from the nine samples that demonstrated a genetic distance of 3%, the total number of sequences, the coverage, and the number of OTUs were shown in Table 2. The rarefaction curves and rank abundance curves were generated using R software (Version 2.15.3). The rarefaction curves tended to approach the saturation plateau, while the rank abundance curves revealed a few dominant taxa and many low-abundance taxa comprising the bacterial community of the golden takin (Fig. 1). Based on ANOSIM analysis, significant differences were observed in bacterial community structure between group A and group B (*P* < 0.05). When analyzing differences between the two groups, the Alpha diversity index and box-plots intuitively reflected the median, degree of dispersion, maximum, minimum and outlier of species diversity within groups. The number of species in group A was lower than that in group B (Fig. 2), and this difference was significant (*P* < 0.05). However the difference of the community diversity and the species distribution uniformity between two groups were not significant (*P* > 0.05).

**Table 2:** Alpha-diversity of the gut microbiota in the nine golden takins in this study

Sample	Observed species	Shannon	Simpson	Chao1	ACE	Goods coverage
J1	1271	8.309	0.991	1342.163	1343.512	0.998
J2	1292	8.203	0.992	1358.65	1399.993	0.997
J3	1299	8.276	0.991	1373.654	1375.156	0.997
J4	1300	8.26	0.991	1394.565	1408.729	0.997
J5	1366	8.394	0.992	1444.019	1456.449	0.997
J6	1495	8.634	0.993	1566.16	1582.534	0.997
J7	1450	8.644	0.994	1532.564	1541.698	0.997
J8	1405	8.428	0.992	1542.244	1520.494	0.996
J9	1289	8.1	0.989	1355.071	1360.503	0.997

### Taxonomic composition

**Fig. 3:**
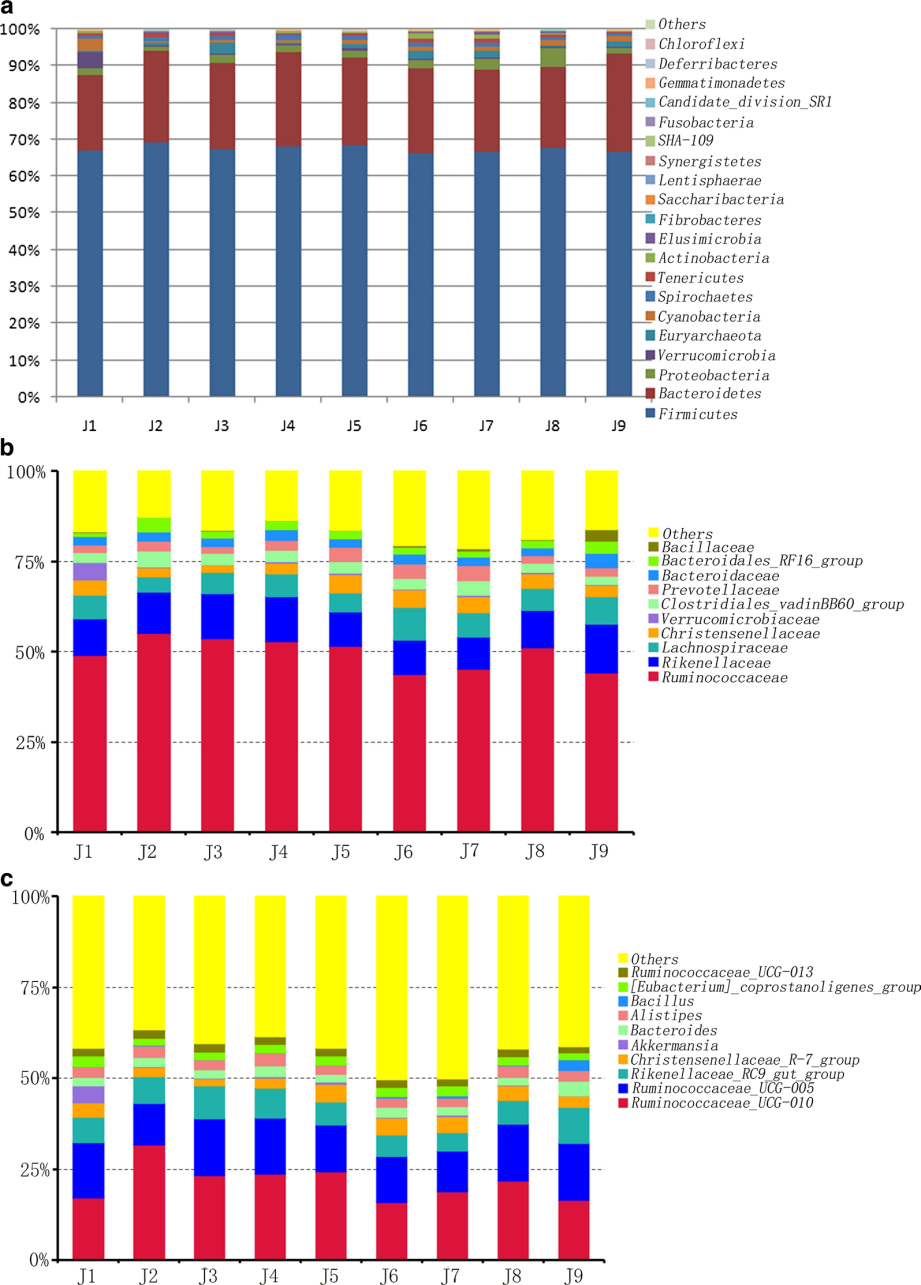
Gut bacterial composition at the phylum level per sample (**a**) and the relative abundance of the top ten biological species at the family level (**b**) and the genus level (**c**)

A total of 20 prokaryotic phyla were identified from the nine golden takins (Fig. 3a). The majority of their gut flora belonged to two phyla: *Firmicutes* (66.37–69.23%) and *Bacteroidetes* (21.26–26.55%). These two phyla represented more than 90% of the total sequences in all nine samples. However, *Euryarchaeota* belonged to the Archaeal domain, and the remainder of the identified bacteria belonged to the bacterial domain. Furthermore, variations were noted in the microbiota among samples, e.g. *Synergistetes* was only found in samples J1, J6, J7, and J8; *Fusobacteria* was only identified in samples J6 and J7; *Gemmatimonadetes* was only observed in sample J7; *Deferribacteres* were identified in sample J6; *Chloroflexi* was only present in J1, J5, and J8.

At the family level, we detected 96 families, and the abundance of unclassified bacteria in the samples was 17.04%. The distribution proportion was greater than 0.5% for 10 families (Fig. 3b). Among these 10 families, *Ruminococcaceae* was predominant, with an abundance of 49.61%, followed by *Rikenellaceae* (10.93%), *Lachnospiraceae* (6.41%), *Christensenellaceae* (3.68%), *Clostridiales_vadinBB60_group* (3.15%), *Prevotellaceae* (2.92%), *Bacteroidaceae* (2.64%), *Bacteroidales_RF16_group* (2.32%), *Verrucomicrobiaceae* (0.79%), and *Bacillaceae* (0.50%). The most abundant families were *Ruminococcaceae*, *Rikenellaceae* and *Lachnospiraceae*, which comprised approximately 66.95% of total sequences.

At the genus level, the percentage of unclassified bacteria averaged 42.52% (ranging from 36.7 to 50.27%), and the remainder were classified into 216 genera. The distribution proportion was greater than 0.5% in 10 genera (Fig. 3c). Among these 10 genera, *Ruminococcaceae_UCG*-*010* was predominant, with an abundance of 21.44%, followed by *Ruminococcaceae_UCG*-*005* (13.93%), *Rikenellaceae_RC9_gut_group* (7.30%), *Christensenellaceae_R*-*7_group* (3.56%), *Alistipes* (2.84%), *Bacteroides* (2.64%), *[Eubacterium]_coprostanoligenes_group* (2.36%), *Ruminococcaceae_UCG*-*013* (2.11%), *Akkermansia* (0.79%), and *Bacillus* (0.50%). Of the 10 genera, *Ruminococcaceae_UCG*-*010*, *Ruminococcaceae_UCG*-*005*, *Christensenellaceae_R*-*7_group*, *Ruminococcaceae_UCG*-*013*, *Bacillus*, and *[Eubacterium]_coprostanoligenes_group* belong to *Firmicutes*. *Rikenellaceae_RC9_gut_group*, *Alistipes* and *Bacteroides* belong to *Bacteroidetes*, and *Akkermansia* belongs to *Verrucomicrobia*.

**Fig. 4:**
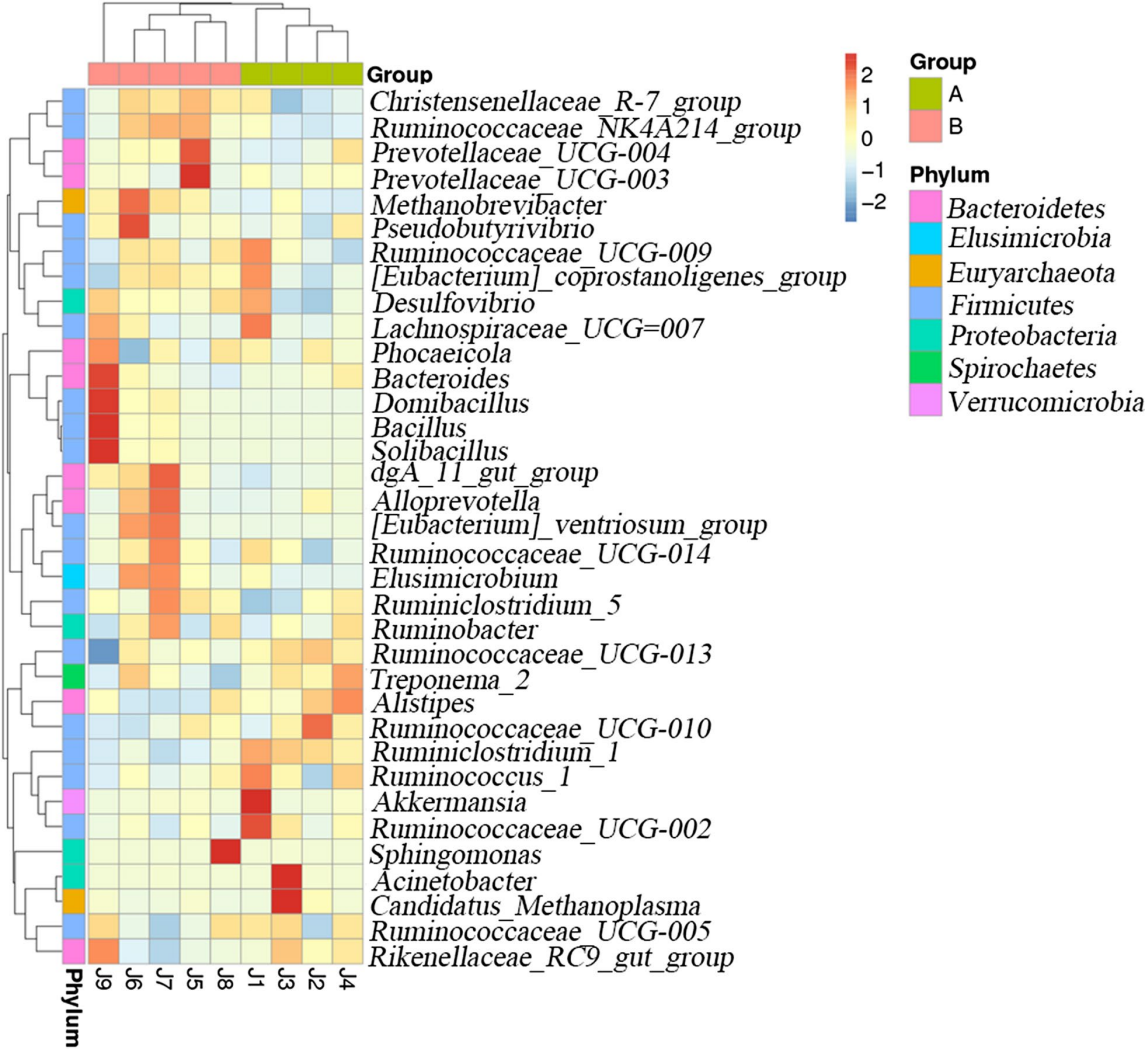
Double dendrogram showing the bacterial distribution among the fecal samples of nine golden takins. The abundances of the top 35 genera were sorted for the analysis. The heatmap plot depicts the relative percentage of each bacterial genus (variables clustering on the y-axis) within each sample (x-axis clustering). The relative values for the bacterial genus are depicted by color intensity in the legend indicated *at the top right* of the figure. Clusters based on the distance of the nine samples along the x-axis and the bacterial genera along the y-axis are indicated in the upper part and *left* of the figure, respectively. *Different colors on the x-axis* represent different groups, and *different colors* on the y-axis represent which phylum they belong to. The meaning of the *different colors* is indicated at the *top right* of the figure

**Fig. 5:**
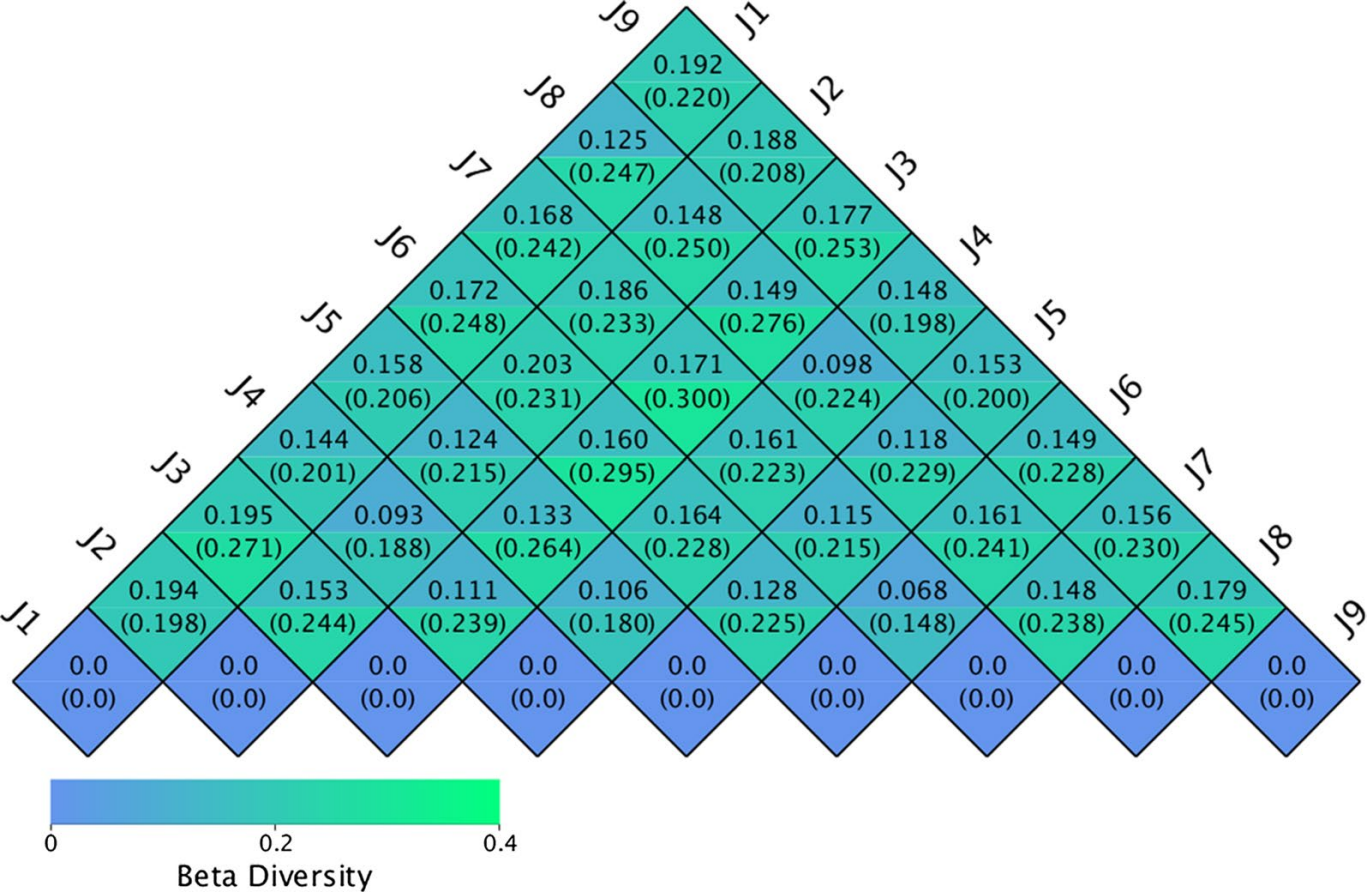
Heatmap of Weighted Unifrac and Unweighted Unifrac distances. In the same square, a fluctuation in two values indicates the Weighted Unifrac and Unweighted Unifrac distances. The number in the diagram pane is the discrepancy coefficient between any two samples. The smaller the discrepancy coefficient between two samples, the smaller the difference in species diversity

**Fig. 6:**
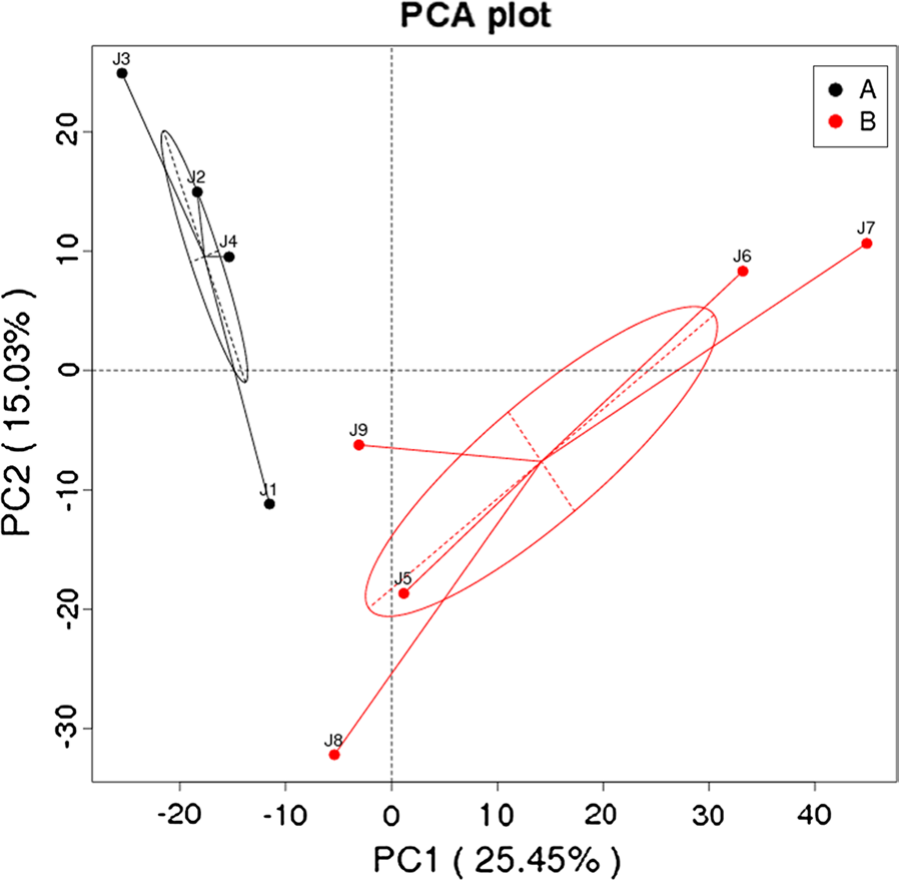
PCA of bacterial community structures of the gut microbiota in the two sample groups. *Each symbol* represents an individual gut microbiota. *Black* and *red symbols* represent the winter group and the spring group, respectively

To know the bacterial community profiles at the genus level, we plotted a clustered heatmap. The result indicated that the sample J1, J2, J3 and J4 (group A) grouped together, while the sample J5, J6, J7 and J8 (group B) clustered together (Fig. 4). To compare the microbial communities across the different samples, we plotted another heatmap based on Weighted Unifrac and Unweighted Unifrac distances (Fig. 5). The similarity of the samples was consistent with the clustering results. In addition, the PCA score plot revealed the separation of group A from group B (Fig. 6), which was consistent with the heatmap results.

### Analysis of species abundance

We performed t test and Mann–Whitney U test to evaluate the significant differences (*P* < 0.05) among species. At the phylum level, *Actinobacteria* was significantly more abundant in group B than in group A. At the class level, *Coriobacteriia* and *Deltaproteobacteria* were significantly less abundant in group A than in group B. At the order level, *Coriobacteriales* was also significantly higher in group B. At the family level, *Coriobacteriaceae* was significantly higher in group B, while *Ruminococcaceae* and *Bacteroidales_S24*-*7_group* were higher in group A (Table 3). Eighteen of the 216 genera were significantly different (*P* < 0.05) between group A and group B (Table 4). Among these 18 genera, *Parabacteroides*, *Sphaerochaeta*, *Ruminiclostridium_1* and *Phascolarctobacterium* were significantly higher in group A, while the others were significantly higher in group B. Additionally, 13 of the 18 genera belonged to *Firmicutes*.

**Table 3:** Seasonal (winter and spring) comparisons for species abundance at the phylum, class, order and family levels

Taxa	Avg (A) (%)	Avg (B) (%)	*P* value	Annotation
*Actinobacteria*	0.13	0.73	0.016	Phylum
*Coriobacteriia*	0.11	0.66	0.016	Class
*Deltaproteobacteria*	0.63	0.88	0.041	Class
*Coriobacteriales*	0.11	0.66	0.016	Order
*Ruminococcaceae*	52.67	47.17	0.039	Family
*Bacteroidales_S24*-*7_group*	0.99	0.33	0.016	Family
*Coriobacteriaceae*	0.11	0.66	0.016	Family
*p*-*2534*-*18B5_gut_group*	0.30	0.75	0.012	Family

**Table 4:** Seasonal (winter and spring) comparisons for species abundance at the genus level

Phylum	Genus	Avg (A) (%)	Avg (B) (%)	*P* value
*Euryarchaeota*	*Methanobrevibacter*	0.239	1.008	0.049
*Actinobacteria*	*Atopobium*	0.046	0.267	0.032
*Actinobacteria*	*Senegalimassilia*	0.012	0.076	0.019
*Bacteroidetes*	*Parabacteroides*	0.050	0.024	0.012
*Spirochaetes*	*Sphaerochaeta*	0.025	0.007	0.007
*Firmicutes*	*Ruminococcaceae_NK4A214_group*	0.513	0.765	0.033
*Firmicutes*	*Ruminiclostridium_1*	0.765	0.366	0.001
*Firmicutes*	*Ruminococcus_2*	0.168	0.397	0.000
*Firmicutes*	*Phascolarctobacterium*	0.367	0.267	0.047
*Firmicutes*	*Acetitomaculum*	0.030	0.101	0.016
*Firmicutes*	*Hydrogenoanaerobacterium*	0.093	0.119	0.046
*Firmicutes*	*[Eubacterium]_nodatum_group*	0.046	0.102	0.011
*Firmicute*	*Marvinbryantia*	0.041	0.076	0.032
*Firmicutes*	*Lachnospiraceae_UCG*-*001*	0.030	0.070	0.005
*Firmicutes*	*Lachnospiraceae_NK4B4_group*	0.025	0.046	0.009
*Firmicutes*	*Solobacterium*	0.005	0.021	0.036
*Firmicutes*	*Family_XIII_UCG*-*001*	0.011	0.022	0.024
*Firmicutes*	*Papillibacter*	0.005	0.014	0.035

## Discussion

In present study, we first performed high-throughput sequencing to characterize the microbial community in the golden takin. Similar to previous researches (Costa et al. 2012; Oikonomou et al. 2013), the golden takin gut microbiota was also dominated by *Firmicutes* (67.59%) and *Bacteroidetes* (23.57%), followed by *Proteobacteria* (2.37%). Firstly, *Firmicutes* was an advantaged group of bacteria in the gastrointestinal tract and fecal flora of various animals (Ley et al. 2008). The advantage exhibited by *Firmicutes* might be related to the anatomical physiology and diet of specific species that feed primarily upon insoluble fiber and in which the cecum and colon were the major sites of fermentation (Costa et al. 2012). Within *Firmicutes*, *Clostridiales* (65.42%) dominated in the feces of golden takins. According to previous studies, *Clostridiales* accounted for 24–59% of human fecal samples and was also the dominant order in the canine gut microbiota; thus, this family was an important index of intestinal bacterial ecosystem function and metabolic differences (Wang et al. 2005). Additionally, *Ruminococcaceae* dominated in the nine golden takins, and *Lachnospiraceae* was present at elevated levels; these bacteria were regarded as fiber-degraders in the herbivore rumen and hindgut (Bian et al. 2013; Jami and Mizrahi 2012). Trace amounts of another major fiber-degrader in the rumen, *Fibrobacter*, were detected in the feces of the nine golden takins. Secondly, *Bacteroidetes* were the second-most predominant components of the gut flora in the nine golden takins, which were consistent with previous researches (Bian et al. 2013; Costa et al. 2012; Oikonomou et al. 2013). Conversely, *Bacteroidetes* constitutes the dominant group in the rumens of other ruminants (Bhatt et al. 2013; Jami and Mizrahi 2012). *Bacteroidetes* degraded high-molecular-weight organic matter, such as protein and carbohydrates, and complemented the host genome to degrade resistant foods such as plant cell wall compounds (including cellulose, pectin, and xylan) (Thoetkiattikul et al. 2013). *Bacteroides* also degraded host sources of carbohydrates, primarily derived from gastrointestinal tract secretions, such as mucins and chondroitin sulfate polysaccharides (Salyers et al. 1977). Additionally, *Bacteroidetes* produces butyrate, which was the product of colonic fermentation. *Bacteroidetes* possessed antitumor properties, which played important roles in maintaining intestinal health (Kim and Milner 2007), and participated in toxic or mutagenic bile acid metabolism and transformation (Smith et al. 2005). In our study, the families *Rikenellaceae*, *Prevotellaceae* and *Bacteroidaceae* dominated within this phylum. Thus, *Bacteroidetes* played an equally important role as *Firmicutes* in the digestive system of herbivorous animals. Thirdly, *Proteobacteria* constituted a large group of bacteria comprising various bacterial taxa that are capable of catabolizing a wide range of feedstuff components (Evans et al. 2010), which advantages these bacteria in the panda (Wei et al. 2012). These differences may be caused by different community structures in the host diet and bowel. In our study, at the genus level, the highest species distribution proportions were observed for *Ruminococcaceae_UCG*-*010* and *Ruminococcaceae_UCG*-*00*, which belonged to *Ruminococcaceae* and were involved in fiber digestion. Fourthly, unclassified bacteria in the nine samples accounted for 18.82–22.48% of sequences. According to previous studies, many animal gut microbiomes contained certain proportions of unclassified bacteria (Bian et al. 2013; Costa et al. 2012; Wu et al. 2016). The reason of this might due to the limited database of 16S RNA gene sequences and little research on classification of fecal microbes. Unclassified bacteria and their functions in different hosts required further research.

The PCA and heatmap analyses indicated that the separation was observed between the microbiota of group A and group B. However, within groups, there was a high percent identity, suggesting the fecal microbiota of golden takins may be influenced by different seasons. Moreover, rarefaction curves revealed lower phylogenetic diversity and numbers of observed species (OTUs) in the winter microbiota than in the spring microbiota. At the same time, the spring microbiota exhibited higher numbers of OTUs with significant differences (Fig. 2). Sun et al. (2016) also found that the dietary shift from winter to spring affected the Tibetan macaque gut microbial composition and diversity. For our research, the diets of the nine golden takins in winter and spring were roughly the same. The significant differences (*P* < 0.05) among species showed that the relative abundances of *Firmicutes* and *Bacteroidetes* in spring were slightly decreased compared with those in winter, *Acidobacteria* differed significantly between the two seasons and was significantly higher in the spring at the phylum level. According to previous studies, *Acidobacteria* was present in many wetland soils and contained a number of genes encoding cellulases and hemicellulases. Thus, these bacteria might play an important roles in plant residue degradation (Kanokratana et al. 2011; Pankratov et al. 2011). At the class level, *Coriobacteriia* and *Deltaproteobacteria* were significantly more abundant in the spring than in the winter. *Coriobacteriia* belonged to *Actinobacteria*, and *Deltaproteobacteria* belonged to *Proteobacteria*. *Actinobacteria* was an important secondary metabolite (enzyme and antibiotics) producer that also played this role in animal intestines, and the secondary metabolites of *Actinobacteria* are potent antibiotics (Bull et al. 2005; Cundliffe 2006). We hypothesized that the rise in *Coriobacteriia* facilitated better resistance to harmful microorganisms external to the intestinal environment, and the rise in *Deltaproteobacteria* was attributable to decreased food quality. Instestingly, 18 genera were significantly different (*P* < 0.05) between spring and winter, and 13 of these 18 genera belonged to *Firmicutes*; among these 13 genera, two genera exhibited higher abundances in the winter than in the spring. In contrast, the other 11 genera were less abundant in the winter than in the spring. Thus, *Firmicutes* might play different roles in different seasons (Carey et al. 2012; Ley et al. 2008; Taglialatela et al. 2009). The weather in spring was highly variable, which was suitable for the proliferation of a variety of pathogenic microorganisms; thus, food might contain greater numbers of pathogenic microorganisms. We suspected that the seasonal differences might be due to the feeding quality, and the intestinal flora of the golden takin might change in the spring to enhance resistance and facilitate the ability to withstand changes in the outside world.

Previous researches also showed that the fecal microbiota of many mammals might be influenced by sex (Bian et al. 2013; Liu et al. 2014; Wu et al. 2016). In present study, the four female golden takins demonstrated highly similar microbiota and the five golden takin males had relatively highly similar microbiota, although J6 and J7 were relatively far from the other three samples, which might indicate that the fecal microbiota of golden takins might also be influenced by the animal’s sex. Sex-related differences might be induced by sex hormones and their effects on gene expression as well as the immune system but also might be attributable to innate physiological differences between males and females (Mcclelland and Smith 2011; Zhao et al. 2013). However, the physiological mechanisms underlying sexual influence were uncertain.

In conclusion, we describe the predominant fecal bacterial populations in golden takin and offered the taxonomic baseline for further research investigating the intestinal ecosystems in golden takins. We also found that the composition of gut microbiota was influenced by the season which might help to strengthen the management of feed quality in the spring to prevent disease in these animals. These observations also increased our understanding of the bacterial ecosystems in this vulnerable animal.

## Abbreviations

DNA: deoxyribonucleic acid
PCR: polymerase chain reaction
PCA: principal component analysis
OTUs: operational taxonomic unit
